# Sensory Attenuation and Agency in Cooperative and Individual Contexts: Exploring the Role of Empathy in Action Perception

**DOI:** 10.3390/brainsci15070688

**Published:** 2025-06-26

**Authors:** Sofia Tagini, Ada Ghiggia, Sara Falco, Lorys Castelli, Alessandro Mauro, Federica Scarpina

**Affiliations:** 1 “Rita Levi Montalcini” Department of Neurosciences, University of Turin, Via Cherasco, 15, 10126 Turin, TO, Italy; sofia.tagini@unito.it (S.T.); alessandro.mauro@unito.it (A.M.); 2 Laboratorio di Ricerca in Neurobiologia Clinica, Ospedale San Giuseppe, IRCCS Istituto Auxologico Italiano, Via Cadorna, 90, 28824 Piancavallo, VCO, Italy; 3 Department of Life Sciences, University of Trieste, Via Licio Giorgieri, 5, 34127 Trieste, TS, Italy; ada.ghiggia@units.it; 4 Department of Psychology, University of Turin, Via Giuseppe Verdi, 10, 10124 Turin, TO, Italy; s.falco@unito.it (S.F.); lorys.castelli@unito.it (L.C.)

**Keywords:** sensory attenuation, agency, empathy, sensorimotor equivalence, social cognition, psychophysics

## Abstract

Background/Objectives: Sensory attenuation refers to the reduced perceptual intensity of self-generated stimuli and is considered a key marker of the sense of agency. While this phenomenon has been widely documented in individual contexts, less is known about how it operates during cooperative actions. In this study, we adopted a psychophysical approach to investigate sensory attenuation for auditory stimuli in both individual and interactive action contexts and examined the role of empathic traits in shaping the experience of agency. Methods: A two-forced choices perceptual judgement task with auditory stimuli was adopted in healthy participants (*n* = 57), who judged the loudness of tones generated either by themselves or another person, across individual and cooperative conditions. To control for the factor of gender that might potentially influence prosocial attitudes, only cisgender women were included in this study. Our findings confirmed sensory attenuation for self-generated sounds in cooperative actions. However, contrary to previous reports, we did not observe enhanced attenuation in interactive contexts; instead, other-generated sounds were perceived as louder when embedded in cooperative actions. Notably, higher levels of empathic concern and perspective-taking were associated with reduced sensory attenuation in individual contexts, suggesting that empathy may modulate perceived self–other boundaries in agency experience. Conclusions: These results challenge the view of sensory attenuation as a strict functional signature of self-agency and support a sensorimotor equivalence model, in which social and psychological variables shape the perception of action outcomes. This evidence is also supported by the convergence of neural networks involved in agency, perspective-taking, and empathy.

## 1. Introduction

Being in control of our actions and their sensory consequences is a fundamental aspect of selfhood [1]. Moreover, it is essential for the execution of effective, goal-directed behaviors [2]. The intuitive ability to distinguish between our actions and those of others is considered a prerequisite for the experience of self-agency [3]. One prominent phenomenon linked to self-agency is sensory self-attenuation, according to which self-generated sensory events are typically perceived as less intense than externally generated ones [4,5]. This perceptual attenuation might serve as a cue to distinguish between self- and other-generated stimuli and is regarded as a proxy for pre-reflective self-agency. Sensory attenuation has been observed across multiple sensory modalities [6] and is commonly explained within the framework of the internal forward models used to predict the sensory consequences of actions [7]. At the neural level, when the actual sensory feedback (i.e., the afferent copy) matches the brain predictions (i.e., the efference copy), the activity in the sensory cortices is reduced; thus, the perceptual experience is attenuated [4,8].

The hypothesis of social sensorimotor *differentiation* posits that the efference copy of a motor command, which is used to predict the sensory consequences of the action, is generated only during voluntary movements. Others’ actions do not trigger such predictive mechanisms, thus, externally generated sensory events cannot find any internal match. In other words, since sensory attenuation is due to a privileged access to the internal motor signals generated only during action execution, it should be specifically related to the sensory consequence of one’s own actions [9]. Then, contributing to the self–other differentiation and to the experience of self-agency. Nonetheless, the specificity of sensory attenuation as a marker of self-agency has been challenged.

Some studies suggest that observed actions—especially those performed by other humans—can be represented in the brain similarly to the executed ones [10,11,12], possibly triggering the same predictive mechanisms engaged during motor execution [13]. The hypothesis of social sensorimotor *equivalence* assumes that internal forward models may be activated also during the observation of actions executed by others [14], which may lead to sensory attenuation even for externally generated sensory events. From a neural perspective, the mirror neurons system (MNS) was extensively identified as cortical underpinning of self–other sensorimotor equivalence [15], which might even anticipate (i.e., fire before) the onset of the observed/executed action, when it can be predicted by contextual cues [16,17]. Also, Krol and colleagues [18] provided evidence supporting the notion that the sensorimotor system, and especially its parietal part, might represent and simulate predictable goal-directed actions, before their actual observation. Accordingly, sense of agency might be informed also by a priori heuristic, regardless of the retrospective correspondence between sensorimotor expectations and outcomes [19,20]. Di Plinio and colleagues [19] demonstrated that the “prospective” sense of agency might be especially triggered in highly predictable contexts, in which the individual learned a consistent association between one’s intention to act, the action execution and outcome; similarly, temporal predictability can also influence the sensory attenuation phenomenon [21]. Taken together, this evidence suggests that action execution might not be necessary to sensory self-attenuation and self-agency. In fact, the intention to produce an outcome might be even more salient than who performed the action, especially when these outcomes are not solely attributed to the self but are co-owned or co-predicted with a partner [18]. To sum up, both hypotheses seem to recognize the key role of predictive models in sensory attenuation. However, the hypothesis of social sensorimotor differentiation exclusively relates perceptual attenuation (i.e., internal forward models) to executed actions, while the hypothesis of social sensorimotor equivalence extends this phenomenon to observed or vicarious actions.

To verify these two theoretical frameworks in the context of sensory attenuation, Weiss and colleagues proposed a two-alternative forced choice auditory task in which participants judged which of two consecutively presented tones was louder [9,22]. Sounds were either self-generated by pressing a button or externally generated by a computer or another person. The results consistently showed reduced perceived loudness for self-generated sounds, independent of the predictability of the external agent’s actions. These findings support the specificity of sensory attenuation for self-generated events. Furthermore, Weiss and colleagues [22] extended their paradigm to cooperative action contexts, reflecting real-life scenarios where actions are socially coordinated. Participants either acted independently or as part of a cooperative dyad (e.g., pressing a button in response to another person’s request, or requesting another person to act). They found that sensory attenuation persisted when participants acted at another’s request, suggesting that self-agency was maintained and even enhanced. However, attenuation also occurred when the participant merely initiated the cooperative sequence (i.e., asked the experimenter to press the button), even though they did not act themselves. The authors interpreted these findings as empirical evidence that self-agency can extend beyond individual action to include socially mediated outcomes. Social interactions may trigger the integration of others into one’s internal sensorimotor representation, linking one’s intention to both the other’s action and its sensory consequences. This proposal raises important questions about the nature of self-agency and its neural markers. Can agency be attributed to outcomes achieved through others’ actions? Does sensory attenuation reflect only self-generated motor commands, or can it emerge from shared sensorimotor representations? While Weiss and colleagues’ findings are compelling, they have yet to be replicated, and the mechanisms underlying stronger attenuation in cooperative contexts remain speculative. In this study, we sought to further explore these mechanisms by adopting the experimental paradigm proposed by Weiss et al. [22] to probe sensory attenuation in both individual and cooperative contexts.

Our goal was to evaluate whether attenuation functions as a marker of pre-reflective self-agency, as predicted by the differentiation account, or whether it extends to observed actions, supporting the equivalence account. Moreover, here we aimed to verify the potential influence of interindividual psychological traits, particularly empathy, on sensory attenuation and the experience of agency in interactive settings. Indeed, social cognition research has demonstrated that perception and action can be modulated by the social environment and the agent’s internal psychological states [23]. Empathy is a complex and multifaceted psychological construct, involving the ability to understand and share others’ emotions while maintaining a self–other distinction. Emotional or affective empathy refers to the possibility of experiencing the feelings of another person as our own [24]. Cognitive empathy is the capacity to recognize and understand others’ emotions, without necessarily “feeling” them [25,26], relying on cognitive mechanisms such as perspective-taking and mentalizing [27]. We speculate that higher expression of empathy might favor interpersonal closeness and self–other convergence, possibly resulting in less differentiation between self-generated and other-generated sounds (i.e., weaker sensory self-attenuation).

## 2. Materials and Methods

### 2.1. Participants

Participants received no remuneration for their participation and were naïve to the experimental procedure; they gave written informed consent before participating in the study. Only right-handed individuals participated in this study. All participants self-reported normal hearing. We included only female cisgender participants (i.e., their gender identity aligns with the sex assigned to them at birth). The specific impact of gender on sensory attenuation has not been addressed in the literature, however, there is substantial evidence of gender differences in various sensory processing, including the perception of loudness [28]; thus, it is plausible for gender to influence sensory attenuation as well. More crucially, gender differences were consistently reported concerning prosocial attitudes and empathy [29]. Furthermore, we wanted to maintain gender equivalence between the participant and the experimenter, who was a cisgender woman, with no close or intimate relationship with the participants. No exclusion criteria were assumed relative to participants’ age or education.

We enrolled 57 participants (average age (SD) in years: 40.93 (16.77); age 20–30: *n* = 28, age 40–50: *n* = 20, age 60 or more: *n* = 9; secondary education: 12.28% (*n* = 7); tertiary education: 43.86% (*n* = 25); academic education: 42.11% (*n* = 24); post-graduate education: 1.75% (*n* = 1).

### 2.2. Procedure

#### 2.2.1. Perceptual Judgment Task

Based on Weiss and colleagues [22], a two-forced choices perceptual judgement task was adopted, in which participants should decide which one of two consecutive tones was “louder”. The experiment was conducted in a quiet room. The participant sits in front of a 16-inch laptop computer with the forearms comfortably placed on the table surface.

**Figure 1:**
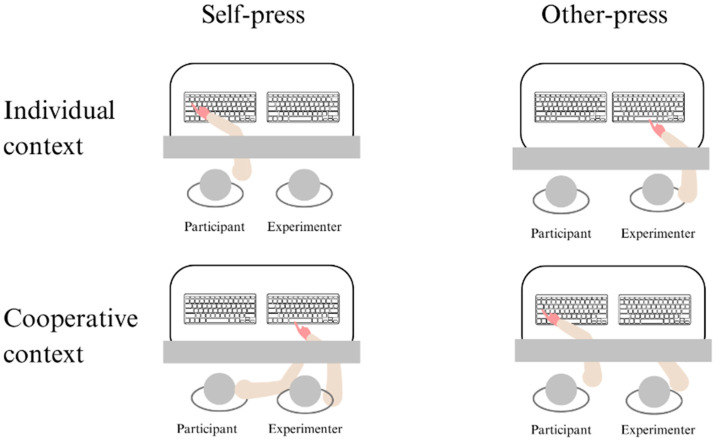
Experimental setting.

As illustrated in Figure 1, the experimenter was sitting right next to the participant, in the same position, in front of a standard keyboard connected to the participant’s laptop. Both the participant and the experimenter could see their hands on the keyboards; however, they could not see their forearms, which were hidden by a horizontal plastic panel. Before starting the test phase, participants performed two acquisition blocks with 5 trials each: in one block, participants pressed the spacebar with their right index finger, and a standard tone of 74 dB was generated after 50 milliseconds (ms); in the other block, they observed the experimenter doing the same action. This phase was introduced to enhance the logical association between the button press action and its auditory consequences.

In the test phase, the spacebar press on the keyboard elicited a 74 dB standard tone, which was followed by a comparison tone randomly selected among 7 possible magnitudes (i.e., between 71 and 77 dB, in 1 dB steps), after a random interval between 800 and 1200 ms. Each comparison tone magnitude was presented 10 times: overall, participants performed 70 trials in each experimental condition in random order across participants. Sound stimuli were sine tones of 1000 Hz lasting 100 ms, presented binaurally through headphones at a constant system volume. In each trial, judgments of loudness were always provided by the participants by pressing the key 1 (“first louder”) or 2 (“second louder”) on the keyboard with their left index finger. The entire experiment was 60 min long. OpenSesame software (version 3.2.8 [30]) was used to run the experiment.

Four experimental conditions were tested in which we modulated both the action context (i.e., individual or cooperative) and the person who pressed the spacebar to generate the tones (i.e., the participant or the experimenter) as follows:

1. *Individual context self-press*: the participant pressed the spacebar with the right index finger, without being involved in any interaction with the experimenter;
2. *Individual context other-press*: the participant saw the experimenter pressing the spacebar with the right index finger, without being involved in any interaction with the experimenter;
3. *Cooperative context self-press*: the participant pressed the spacebar with the right index finger, whenever the experimenter requested her to do so by touching the participant’s right forearm with her right hand, which was occluded to prevent the visual anticipation of touch;
4. *Cooperative context other-press*: the experimenter pressed the spacebar with the right index finger whenever the participant requested her to do so by touching the experimenter’s right forearm with her right hand, which was likewise occluded (as before, to avoid any visual anticipation of touch).

The four experimental conditions were presented in random order between participants. Instructions were provided before each experimental condition on a 16-inch screen laptop computer in front of the participant, specifying who had to press the spacebar and whether the action scenario was individual or cooperative. After the standard tone and the comparison tone were produced, a prompt on the laptop screen appeared, encouraging the participant to judge which of the two sounds was louder. Once the participant answered, a black cross appeared in the middle of the screen, anticipating the “GO” signal. The “GO” signal indicates the beginning of a new trial: as the “GO” signal appears on the screen the agent can act by pressing the spacebar or touching the other’s arm, depending on the experimental condition.

#### 2.2.2. Empathy

The Interpersonal Reactivity Index—IRI [31,32] is a self-report questionnaire about empathy, comprising 28 items answered on a 5-point Likert-type scale ranging from 1 (does not describe me well) to 5 (describes me very well). The questionnaire measures four aspects of empathy. Two are mainly related to the *emotional* component of empathy: including (i) empathic concern (IRI-EC), as measure of individual feelings of warmth and compassion towards others undergoing negative experiences and (ii) personal distress (IRI-PD), which measures self-oriented feelings of anxiety and discomfort that occur in response to others’ distress. On the *cognitive* side, (iii) perspective-taking (IRI-PT), measures the spontaneous tendency to adopt the psychological point of view of others including thoughts and feelings while iv) fantasy (IRI-F) refers to the individual tendency to transpose themselves imaginatively into the feelings and actions of fictitious characters in books, movies, and plays. Each of the four IRI subscales includes 7 items (total scores range from 1 to 35): the higher the score, the higher the expression of empathy. Participants always completed the questionnaire after the experimental task, to avoid any biases.

### 2.3. Statistical Analysis

#### 2.3.1. Perceptual Judgment Task

We performed experimental data pre-processing, although this procedure was not described in the seminal article by Weiss and colleagues [22]. First, we checked for anomalous response times: trials in which the response time was shorter than 50 ms were excluded. This interval is generally considered too short to allow the processing of auditory stimuli [33]. Then, we looked for response times higher than twice the standard deviation from the mean, relative to each participant in each experimental condition: drops in attention may result in such long response times. The main analyses included only valid trials.

Then, we computed the number of times each comparison tone was judged as “louder” to the total number of judgments (i.e., the proportion of “second tone louder” answers), for each participant and condition. Then, a logistic fitting was applied to the observed proportions of “second tone louder” answers in each condition, according to a maximum-likelihood procedure. In addition to the procedure reported in the work of Weiss and colleagues [22], we tested the goodness of each participant’s fit according to the proportion of variance explained by the model (r2). The tone magnitude judged as louder than the standard tone in 50% of trials was taken as the point of subjective equality (PSE); that is, the intensity of sound the participant perceived as equal to the standard tone. This computation implies that behavioral observations were distributed according to the predicted model. Thus, participants with a non-fitting distribution of observations (*p* > 0.05) at least in one experimental condition were excluded from successive analyses. A PSE lower (i.e., less loudness) for self-generated than other-generated sounds suggested the presence of sensory self-attenuation.

Mean and standard deviation were computed for the PSE in the four experimental conditions. The normality of distributions was probed with Shapiro–Wilk’s normality tests; values ± 1.5 times the interquartile range (IQR) were marked as possible outliers. Formal comparisons between the computed PSE with the standard tone loudness (74 dB) were assessed with one-sample t-tests, in the four conditions separately; Bonferroni’s adjustment for multiple comparisons was adopted (alpha _adj_ = 0.0125).

In line with the statistical model described in the original article [22], the PSE values were entered into a repeated measures analysis of variance (ANOVA) with the two within-subject factors of button press (self-press versus other-press) and action context (individual versus cooperative). Here, post hoc comparisons were carried out using estimated marginal means and Bonferroni’s correction for multiple comparisons.

#### 2.3.2. Empathy

Raw scores relative to the IRI questionnaire were used in the statistical models. To explore the role of empathy in association with sensory attenuation, for each participant we computed a ratio index, in both the individual (Individual ratio) and cooperative (Cooperative ratio) context, according to the following formula:

(PSE for other-generated sounds/PSE for self-generated sounds)

These indices are informative of the magnitude of sensory self-attenuation: values higher than one indicate lower PSE (i.e., perceived tone loudness) for self-generated stimuli than other-generated stimuli; thus, the greater the index the higher the sensory self-attenuation effect. Values below one suggest the opposite pattern: the lower the index, the weakest the sensory attenuation effect.

Then, we computed Spearman’s coefficient of correlation between the Cooperative ratio and Individual ratio indices and each of the four components of the IRI (i.e., perspective-taking, fantasy, empathic concern, and personal distress) to test the possible link between the sensory attenuation and the individual expressions of empathy, in the individual and the cooperative context. Since we had a directional hypothesis (i.e., higher expressions of empathic components might be related to a weaker sensory self-attenuation effect because of increased self–other sensorimotor equivalence), we used one-tailed tests.

## 3. Results

### 3.1. Perceptual Judgment Task

Overall, we removed 46 trials with a response time lower than 50 ms, corresponding to 0.29% of total trials; at the individual level, the number of excluded trials ranged from 0 to 2 in each condition, with a maximum of 3 trials excluded in a single participant. On the remaining trials, we identified response times higher than twice the standard deviation from the mean, relative to each participant in each experimental condition. Overall, 4.31% (*n* = 686) were excluded; at the individual level, the number of excluded trials ranged from 1 to 6 when considering the four experimental conditions separately, while the average number of excluded trials in each participant across the entire experimental procedure ranged from 1.5 to 4.75. Interestingly, 21.87% of the excluded trials (i.e., shorter and longer the defined bounds) occurred between the first and the fourth trial, suggesting participants’ unpreparedness for the new experimental block; 78% of invalid trials were distributed over the remaining 66 trials, possibly due to drops in attention or increasing fatigue.

Thirteen participants were excluded because of an anomalous distribution of observations hampering the computation of the PSE, at least in one of the four experimental conditions, and resulting in an insufficient goodness. Overall, 44 participants were included in the final analyses (average age (SD): 42.60 (16.62); secondary education: 15.91% (*n* = 7); tertiary education: 38.64% (*n* = 7); academic education: 43.18% (*n* = 19); post-graduated education: 2.27% (*n* = 1)).

**Figure 2:**
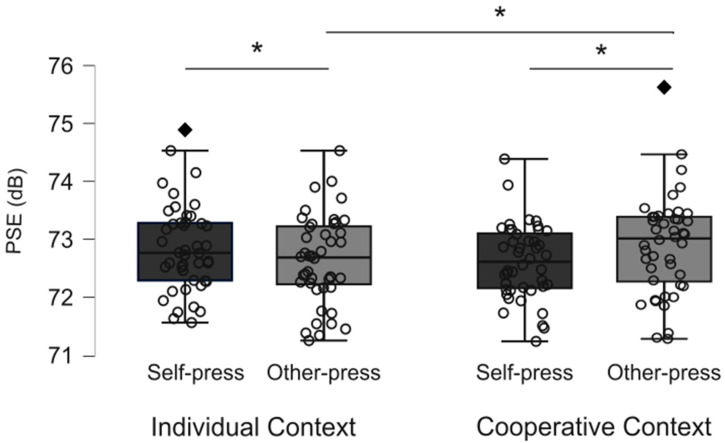
The figure describes the distribution of observations relative to the PSE for the self-press (dark boxes) and other-press (light boxes) conditions in the Individual and Cooperative action contexts. Boxplot bounds represent the 75th (upper bound) and the 25th (lower bound) percentile of each distribution, the horizontal line indicates the median of distribution; whiskers illustrate the minimum (lower fence) and maximum (upper fence) value of ±1.5 × IQ range: diamonds evidence outlier observations (i.e., values outside the ±1.5 × IQR). Significant differences according to post hoc comparisons are indicated with asterisks.

The mean and standard deviation relative to the PSE in the four experimental conditions are reported in Table 1. Observations were normally distributed in each experimental condition (all Shapiro–Wilk’s *p* > 0.05). As illustrated in Figure 2, two independent observations were identified as possible outliers: extreme PSE values were reported in the cooperative action context other-press and individual action context self-press condition, relative to two different participants. After checking for possible data entry and computational anomalies in determining the observed outliers, we included both participants in the main analyses.

As illustrated in Table 1, the PSE in the four experimental conditions was significantly lower than the real standard tone (74 dB), suggesting a generalized reduction in the perceived loudness compared to the real one.

According to the results from the ANOVA, the main effect of action context [F(1,43) = 0.13; *p* = 0.71; η_p_^2^ = 0.003] and the main effect of button press [F(1,43) = 0.06; *p* = 0.8; η_p_^2^ = 0.001] were not significant. More crucially, we observed the significant interaction of Action Context * Button Press [F(1,43) = 10.354; *p* = 0.002; η_p_^2^ = 0.19]. As reported in Figure 2, post hoc comparisons showed that in the individual action context (Figure 2, left panel), the PSE for the self-press button condition (PSE = 72.85) was significantly higher (*p* = 0.024) than in the other-press condition (i.e., when the experimenter produced the sound, PSE = 72.63). Crucially, this pattern was opposite to the expected sensory attenuation effect, according to which a lower PSE for self-generated stimuli compared to other-generated stimuli should be observed. In the cooperative context (see Figure 2, right panel), we observed that the PSE in the self-press condition (PSE = 72.65) was significantly lower compared to the other-press condition (PSE = 72.90, *p* = 0.024), suggesting the presence of the sensory self-attenuation phenomenon.

Then, considering the button press factor, the PSE for self-generated stimuli (i.e., in the self-press condition) was not significantly different between the individual and cooperative action contexts (*p* = 0.056). Instead, the PSE for the other-generated stimuli (i.e., in the other-press condition) in the cooperative action context was significantly higher than in the individual context (*p* = 0.029).

For clarity’s sake, in Supporting Information Table S1, we reported the ANOVA results excluding the two outliers detected from the sample. Interestingly, we confirmed the significant interaction Action Context * Button Press [F(1,41) = 8.27; *p* = 0.006; η_p_^2^ = 0.17]; however, post hoc comparisons showed that the difference between the PSE for self-generated compared to other-generated sounds was statistically significant in the cooperative action context but not in the individual context. Thus, the sensory attenuation for self-generated sounds was consistently observed only in the cooperative action context; on the contrary, in the individual action context, self-generated and other-generated sounds were perceived as equally loud.

A post hoc analysis was performed with G*Power software (version 3.1.9.6) [34] to compute the achieved statistical power relative to the 44 participants included in our main analysis (i.e., within-subjects repeated measures ANOVA) and specifically to the observed significant interaction of Action Context × Button Press. Given the observed effect size (η_p_^2^ = 0.19) and an average strong correlation between repeated measurements (r = 0.568), assuming a 0.05 alfa, the power was 0.99.

### 3.2. Empathy

**Figure 3:**
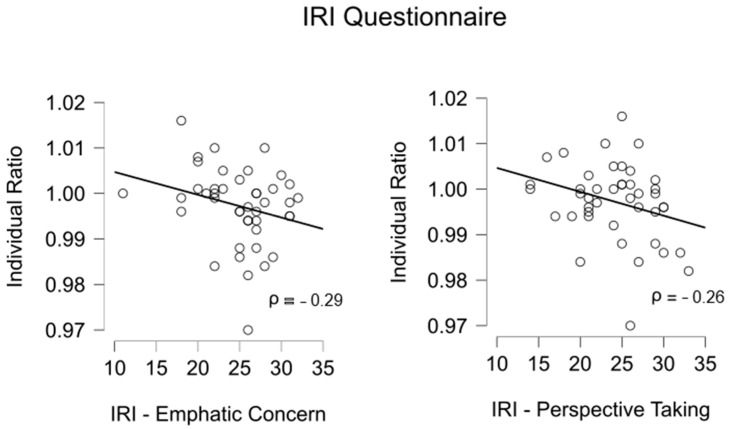
Relationship between the Individual ratio (x-axis) and the Interpersonal Reactivity Index (IRI) (y-axis) scores for the components of empathic concern (**left part**) and perspective-taking (**right part**); *n* = 43.

In Table 2, we report the raw scores relative to the four IRI subscales; in the Supporting Information Table S2, we provide means and standard deviations adjusted according to norms relative to the Italian population [35]. Overall, only one participant reported a score lower than the critical cut-off, relative to IRI Empathic Concern subscale. In Table 3, we reported the results relative to the computation of Spearman’s coefficients and the relative one-tailed *p*-values. Focusing on the cooperative context (Cooperative ratio), no significant results emerged, suggesting that the sensory attenuation phenomenon in the cooperative context was not related to the individual expressions of empathy. This result was in disagreement with our hypothesis. On the other hand, when we considered the individual context (Individual ratio), the empathic concern and perspective-taking scores from the IRI questionnaire were significantly (even if weakly) related to the Individual ratio. As reported in Table 3, the coefficients were negative; thus, as shown in Figure 3, the higher the individual expression of empathic concern, the lower the index, suggesting a weaker sensory self-attenuation effect.

## 4. Discussion

This study provides novel evidence about sensory attenuation of auditory stimuli in individual and cooperative action contexts by adopting Weiss and colleagues’ psychophysical paradigm [22]. When comparing the perceived loudness of self-generated and externally generated sounds, we confirmed the occurrence of sensory attenuation for self-generated sounds in the cooperative action condition [22] since participants perceived the auditory outcome of their actions as attenuated compared to the same sounds generated by the experimenter. While sensory attenuation has traditionally been examined in individual contexts, in line with Weiss et al. [22], our results support the presence of this effect in social cooperation. However, contrary to their findings, we did not observe *enhanced* attenuation in cooperative compared to individual contexts since no differences were observed in the perceived loudness of self-generated sounds between the two action scenarios. Moreover, in our study, other-generated sounds were perceived as louder during cooperative actions than in individual contexts, contrasting Weiss et al.'s report [22] of reduced loudness across both self- and other-generated sounds in cooperation. However, our findings align with more recent neurophysiological [36] and fMRI [37] studies, which point to a *reduction* in the sense of agency during cooperative actions. Beyer et al.'s [36] observed reduced self-agency on the outcome of a computer game (both subjectively in terms of participant’s perceived control and objectively, as neural responses to the game outcomes) when participants rely on a co-player than playing alone. Also, this effect was replicated in their neuroimaging study [37], which linked reduced agency in social contexts to increased activity in the precuneus. The interplay between sensory perception and social engagement might be intricated [38], thus, requiring further investigation of the underlying mechanisms.

Even more unexpectedly, in the individual context, sounds produced by the experimenter were perceived as attenuated compared to self-generated sounds. This effect was not significant after outlier removal; however, this evidence challenges the prevailing notion that sensory attenuation is exclusive to self-performed actions, contributing to the ongoing debate regarding the mechanisms of sensory attenuation and its role as a functional marker of self-agency. The theory of social sensorimotor differentiation posits that sensory attenuation reflects privileged access to internal motor signals, and should therefore only be observed in response to self-generated actions [9]. However, our data suggest that others’ actions may be processed similarly to one’s own, in support of a sensorimotor equivalence. This idea is further supported by Khalighinejad et al. [39], who proposed that observed actions can extend individual agency, making outcomes more salient than the acting agent, even in the absence of explicit interaction. In this study, the act of “touching the arm” may introduce social cues that go beyond a purely instrumental signal, establishing a sensorimotor link between the two individuals’ actions and their consequences, aligning with the concept of shared intentionality [40]. Indeed, Weiss et al. [22] argued that if an action is performed at the request of another person, the initiator may be incorporated into the internal model of action, producing attenuation for externally generated sounds. In other words, when individuals engage in cooperative actions with shared goals, they might form a joint representational space based on overlapping cortical sensorimotor representations for both the observed and executed actions [14]. Thus, the uniquely human capacity to engage in collaborative activities and to form shared intentions might influence how actions are planned and how sensory consequences are perceived. However, these interpretations challenge the assumption that sensory attenuation exclusively reflects self-executed action. Instead, they suggest that the perceived control over the outcomes may matter more than motor execution [39], particularly in cooperative settings where control and execution are shared. In these scenarios, the predictability and timing of triggers and outcomes seem crucial [14,19]: thus, future experiments might control the time between the touch and the action execution, as it might affect the agency feeling.

Further, we examined whether individual psychological traits—specifically empathic dispositions—might modulate sensory attenuation. Our result supports the notion that individual differences in empathy could influence the degree of self–other overlap, even though they may be considered preliminary since associations were weak and our results might not remain significant after applying Bonferroni’s adjustments for multiple comparisons. In the individual context, higher levels of perspective-taking and empathic concern were associated with a smaller difference between perceived loudness of self- vs. other-generated sounds. In other words, the higher the expression of empathic traits the more similar might be the perceived loudness for self-generated and other-generated sounds, possibly because individuals with higher expression of both emotional and cognitive empathy may feel more psychologically attuned to the experimenter, reducing the interindividual perceptual distinction. In fact, some of the participants with the highest expression of empathic traits even reported attenuated loudness for other-generated sounds compared to self-generated sounds, pointing to a more pronounced sensitivity towards others’ than their own actions. However, this link was not observed only in the cooperative condition, in which the shared intentionality may have reduced the need to infer or empathize with the experimenter’s. As Weiss et al. [22] proposed, social interaction itself may promote the inclusion of the other in one's sensorimotor representations. We note, however, that the task used in this study was not emotionally charged. It remains to be seen whether empathy plays a greater role in contexts with higher emotional or social significance. Such an approach may offer insights into perceptions of responsibility in group-based actions, such as bullying or collective violence. 

Then, some methodological considerations must be addressed. First, our sample consisted exclusively of cisgender women, as gender is known to influence prosocial behaviors such as empathy [29]. While this choice aimed to reduce variability, it limits the generalizability of our findings to men. Also, we did not adopt exclusion criteria based on participant’s age or schooling level: education is not expected to affect basic sensory perception but sensory self-attenuation might increase with age due to increasing reliance on predictive models than sensory inputs [41,42] to compensate for decreasing sensory accuracy [43] (but see also [44]). Furthermore, empathy might be affected by age, even though the detrimental effect of aging might be more pronounced on the cognitive than the affective component [45]. Indeed, demographic dissimilarities in terms of gender composition and age of participants between our sample and Weiss and colleagues’ (i.e., which included younger women and also men) might explain the differences observed in the results. About age, evidence about sensory attenuation mechanisms in healthy controls with a wider age-range seems crucial for future studies aimed at investigating this topic in individuals affected by neurodevelopmental motor disorders or psychopathologies, whose onset is typically set later than early adulthood. Furthermore, we did not control for the environmental noise level (e.g., by using acoustic testing procedures or anechoic chambers); this was in line with the previous studies, however, future investigations might take this issue into consideration. Finally, this study employed a substantially reduced number of trials compared to Weiss et al. [22], who used 700 trials across two sessions. This choice might have impacted the statistical power and measurement precision, even though we replicated sensory attenuation effects in the cooperative context. Also, the reduced number of trials might affect the possibility of a good fitting, possibly explaining why several of our participants were excluded because of not enough goodness of fit. In this regard, we note that no information is provided about how (and if) raw data were screened and, eventually, cleaned in the original work by Weiss and colleagues, and whether the goodness of the fitting procedure was tested, limiting the comparison of the two procedures. Nonetheless, the availability of a shorter task might be crucial, especially for clinical applications where participant fatigue, attention fluctuations, and reduced compliance may necessitate shorter sessions. Our findings suggest that a shorter version of the Weiss and colleagues’ [22] paradigm may be suitable for use in clinical populations, especially if combined with a comprehensive assessment of those psychological dimensions possibly shaping agency in social contexts (such as empathy, but also personality traits and other interpersonal attitudes).

From a neural perspective, a recent meta-analysis of fMRI studies identified two major networks supporting the internal forward model of motor control, which is the key mechanism underlying both sensory attenuation and the sense of agency—regardless of whether sensorimotor differentiation or equivalence is assumed [6]. The “feedforward” network is responsible for generating predictions about the outcomes of voluntary actions and comparing them with incoming sensory feedback. Within this network, the supplementary motor area and premotor cortex contribute to initiating actions and forming predictive models based on motor commands, with the cerebellum playing a central coordinating role. Complementarily, the inferior parietal lobule, medial prefrontal cortex, and temporoparietal junction are thought to evaluate the match between predicted and actual outcomes, enabling the distinction between self- and other-generated actions [46,47]. In contrast, the “feedback” network, which includes the superior and middle temporal gyri and the insula, exhibits heightened responsiveness to externally generated stimuli. Crucially, empathy engages brain systems involved in perspective-taking, emotional mirroring, and mentalizing, which partially coincide with these networks. A recent meta-analysis [48] highlighted the medial prefrontal cortex as central to mental state attribution, while the temporoparietal junction and superior temporal sulcus were implicated in both perspective-taking and agency attribution, potentially acting as hubs where social cognition converges with sensorimotor processes. Furthermore, the anterior insula and anterior cingulate cortex process emotional salience and subjective feeling states, while the inferior frontal gyrus and inferior parietal lobule, key nodes of the mirror neuron system, support the simulation of others’ actions and emotions. 

According to the sensorimotor equivalence hypothesis, observing another’s actions may engage internal models similar to those used for executing one’s own actions—thus blurring the distinction between self and other at both motor and perceptual levels. The mirror neuron system, primarily situated in the inferior frontal gyrus and inferior parietal cortex, is activated during both action execution and observation, further supporting this shared representational framework. Altogether, these interconnected neural networks suggest a neurocognitive continuum in which sensory attenuation and agency attribution are not solely anchored in the self, but are modulated by how individuals perceive and relate to others. Within this framework, higher empathy may influence sensorimotor equivalence, even resulting in a higher sensitivity and silence to other- compared to self-generated actions.

## 5. Conclusions

In conclusion, our findings raise important questions about whether sensory attenuation is uniquely tied to self-performed actions and can be considered a functional marker of self-agency. The current evidence does not support a definitive answer. However, our results are more consistent with a model of sensorimotor equivalence, particularly in interactive contexts. This interpretation broadens the scope of sensory attenuation as a proxy for general agency, rather than self-agency. Future research might include experimental conditions where expectations are violated—for example, when the experimenter fails to act upon a participant’s request—to further investigate the role of prediction, control, and social cues in shaping the sense of agency.

## Figures and Tables

**Table 1:** Descriptive statistics are reported relative to the PSE (dB) in the four experimental conditions and the final sample (*n* = 44); Shapiro–Wilks’ statistics are illustrated to inform about the normality of distributions; statistics about one-sample *t*-tests are provided relative to the comparison between the computed PSE and the standard tone loudness (74 dB).

	**Mean dB (SD)**	**Min**	**Max**	**Shapiro–Wilk’s**	**One-Sample *t*-Tests**
**Individual context**					
*Self-press*	72.85 (0.76)	71.56	74.89	0.97, *p* = 0.42	t_43_ = 11.85, *p* < 0.001, d = 1.51
*Other-press*	72.63 (0.76)	71.25	74.53	0.98, *p* = 0.58	t_43_= 10.04, *p* < 0.001, d = 1.79
	**Mean dB (SD)**	**Min**	**Max**	**Shapiro–Wilk’s**	**One-Sample *t*-Tests**
**Cooperative context**					
*Self-press*	72.65 (0.65)	71.28	74.43	0.98, *p* = 0.58	t_43_= 13.83, *p* < 0.001, d = 2.09
*Other-press*	72.90 (0.86)	71.28	75.63	0.96, *p* = 0.13	t_43_= 8.49, *p* < 0.001, d = 1.28

**Table 2:** Average raw scores, standard deviation (SD), minimum, and maximum values for the four IRI subscales in our sample (*n* = 43).

IRI	Mean	SD	Min	Max
*Fantasy*	20	4.4	12	29
*Emphatic Concern*	25	4.35	11	32
*Perspective-Taking*	24.2	4.65	14	33
*Personal Distress*	16.9	4.91	6	27

**Table 3:** Spearman’s coefficients of correlation (ρ) between the Individual ratio and Cooperative ratio and the four IRI subscales are reported. One-tailed *p*-values are illustrated: significant correlations are reported in bold.

	Interpersonal Reactivity Index (IRI)							
	IRI-FS		IRI-EC		IRI-PT		IRI-PD	
	ρ	*p*-Value	ρ	*p*-Value	ρ	*p*-Value	ρ	*p*-Value
**Individual ratio**	0.04	0.60	−0.29	**0.030**	−0.26	**0.045**	0.23	0.93
**Cooperative ratio**	−0.03	0.44	−0.08	0.300	0.12	0.770	−0.09	0.29

## Data Availability

The data that support the findings of this study are available upon reasonable request in Zenodo.org repository at http://doi.org/10.5281/zenodo.11180919.

## Supplementary Materials

The following supporting information can be downloaded at: https://www.mdpi.com/article/10.3390/brainsci15070688/s1, Table S1 Mean, standard deviation and post-hoc statistics are illustrated by Button Press and Action Context. Significant differences are reported in bold; Bonferroni’s correction for multiple tests was adopted; Table S2: Means and standard deviations are reported for our sample (*n* = 43) relative to the four IRI subscale scores, adjusted by age, sex, and education according to Italian normative data [35].
