# Supplementary material for: Sensory Attenuation and Agency in Cooperative and Individual Contexts: Exploring the Role of Empathy in Action Perception
Source: Brain Sci. 2025 Jun 26;15(7):688. doi: 10.3390/brainsci15070688 (PMC12293617; doi:10.3390/brainsci15070688)
Supplement: Supplementary file 1 [file brainsci-15-00688-s001.zip › brainsci-3626665-supplementary.pdf]

Sensory Attenuation and Agency in Cooperative and Individual Contexts: Exploring the Role of Empathy in Action Perception

Supporting Information

S1. Supplementary analyses and results

When preprocessing PSE values, two independent observations were identified as possible outliers in the cooperative action context other-press and individual action context self-press conditions. Here we reported the ANOVA results when excluding these two outlier observations from the sample (n = 42). According to the results from the ANOVA, the main effect of *Action Context* [$F(1,41)$= 0.06; p = 0.81; η_p^2 = 0.001] and the main effect of *Button Press* [$F(1,41)$= 0.06; p = 0.59; η_p^2 = 0.007] were not significant. However, we observed the significant interaction of *Action Context* * *Button Press* [$F(1,41)$= 8.27; p = 0.006; η_p^2 = 0.17]. Average PSE, standard deviations, and post-hoc comparisons are reported in Table S1. We observed that self-generated sounds were perceived as significantly louder in the individual compared to the cooperative context; more crucially, we observed that sounds in the self-press condition were louder than in the other-press condition in the *Cooperative* action context, in line with the sensory attenuation phenomenon, but no significant differences were observed between self and other-generated sounds in the *Individual* action context.

Table S1. Mean, standard deviation and post-hoc statistics are illustrated by *Button Press* and *Action Context.* Significant differences are reported in bold; Bonferroni's correction for multiple tests was adopted.

**Post-hoc comparisons by *Button Press***							
	**Individual**	***vs***	**Cooperative**				
				**Δ Mean**	**SE**	**t**	**Cohen's d**
Self-press	**72.76 (0.65)**		**72.59 (0.59)**	**-0.21**	**0.1**	**-2.10**	**-0.30**
Other-press	72.61 (0.75)		72.82 (0.76)	0.17	0.1	1.74	0.25

**Post-hoc Comparisons by *Action Context***							
				**Δ Mean**	**SE**	**t**	**Cohen's d**
	**Self-press**	***vs***	**Other-press**				
Individual	72.76 (0.65)		72.61 (0.75)	0.15	0.097	1.57	0.22
Cooperative	**72.59 (0.59)**		**72.82 (0.76)**	**-0.23**	**0.097**	**-2.37**	**-0.33**

In Table S2 we provided the mean and standard deviation for the four IRI scores adjusted according to Italian norms (Maddaluno et al., 2022). Also, we showed the number of participants according to the normative cut-off. By convention, scores equal to "zero" are considered clinically relevant; overall, only one participant reported a score lower than the critical cut-off, relative to the IRI *Empathic Concern* subscale.

Table S2. Means and standard deviations are reported for our sample (n = 43) relative to the four IRI subscale scores, adjusted by age, sex, and education according to Italian normative data.

**Interpersonal Reactivity Index**	**Mean**	**SD**	**Participants (*n*) by equivalent score-levels**				
			**0**	**1**	**2**	**3**	**4**
Fantasy	18.63	4.36	0	0	4	10	29
Emphatic concern	23.36	4.39	1	0	2	5	35
Perspective taking	23.06	4.65	0	0	3	2	38
Personal distress	15.82	4.86	0	2	3	4	34
